# Treatment of Acute Tonsillitis

**Published:** 1886-11

**Authors:** 


Treatment of Acute Tonsillitis.—Dr. John Brown states, in the *British Medical Journal*, that it is a rare event for suppuration to occur in acute tonsillitis, if treated early with the following mixture :

| | | |
|---|---|---|
| ℞ | Sodii Salicylat, - - - - | ʒ iss. |
| | Potass. bicarb., - - - - | ʒ iss. |
| | Tinct. Aconit, - - - - | M xl. |
| | Liq. Opii Sed., - - - - | ʒ ss. |
| | Sp. Chloroform, - - - - | ʒ ii. |
| | Aq. Ad., - - - - - | ℥ viii. M. |

Sig. :—℥i-ii every two or three hours for the first thirty-six hours. The same mixture is his sheet anchor in rheumatic fever.—*Med. Age.*

---

During the past summer the infant mortality in New York city increased about fifteen per cent. over that of the summer of 1885, although the city was never in better sanitary condition. This increase in the mortality rates is attributed to the fact that no summer corps of physicians was appointed to visit and prescribe for the sick children of the tenement-house population, as has been the custom heretofore. The Board of Apportionment, having an attack of "economy," refused to appropriate the usual $10,000 for this purpose, and as a result the city has been obliged to expend about this amount for burial expenses of the poor.

